# Elevated Expression of Chemokine CXCL13 in Chronic Hepatitis B Patients Links to Immune Control during Antiviral Therapy

**DOI:** 10.3389/fimmu.2017.00323

**Published:** 2017-03-23

**Authors:** Chao Liu, Xuan Huang, Melanie Werner, Ruth Broering, Jun Ge, Yongyin Li, Baolin Liao, Jian Sun, Jie Peng, Mengji Lu, Jinlin Hou, Xiaoyong Zhang

**Affiliations:** ^1^State Key Laboratory of Organ Failure Research, Guangdong Provincial Key Laboratory of Viral Hepatitis Research, Department of Infectious Diseases, Nanfang Hospital, Southern Medical University, Guangzhou, China; ^2^Department of Gastroenterology and Hepatology, Essen University Hospital, University of Duisburg-Essen, Essen, Germany; ^3^Department of Infectious Disease, Guangzhou Eighth People’s Hospital, Guangzhou Medical University, Guangzhou, China; ^4^Institute of Virology, Essen University Hospital, University of Duisburg-Essen, Essen, Germany

**Keywords:** hepatitis B virus, chronic hepatitis B, CXCL13, Kupffer cell, hepatitis B virus e antigen seroconversion

## Abstract

C–X–C-chemokine ligand 13 (CXCL13), the ligand for C–X–C chemokine receptor type 5 (CXCR5), is a major regulator of B-cell trafficking and plays an integral role in age-dependent clearance of hepatitis B virus (HBV) in the mouse model. However, the expression and function of CXCL13 in patients with chronic hepatitis B (CHB) remain unknown. By use of liver cell subpopulations isolated from CHB patients, we found that CXCL13 mRNA was abundantly expressed in Kupffer cells (KCs), but not in primary hepatocytes, liver sinusoidal endothelial cells, and hepatic stellate cells. Interestingly, KC isolated from HBV-positive liver had much higher level of CXCL13 expression than non-HBV-infected controls. And its expression was induced by toll-like receptor 3 ligand poly I:C stimulation. Moreover, intense expression of CXCL13 protein and accumulation of CD4^+^ T and B cells were evident in follicular-like structures in the liver tissue of CHB patients, which indicated its chemotactic effect on CXCR5^+^ CD4^+^ cells and B cells. Consistently, the levels of serum CXCL13 were significantly higher in the CHB patients than in healthy controls. Furthermore, CXCL13 concentration was increased in the complete response (CR) group during weeks 0–12 and did not change significantly during the course of telbivudine treatment, compared with the patients who didn’t achieve CR. In conclusion, the HBV-related increase of CXCL13 production in KC and serum CXCL13 level during telbivudine treatment might be associated with immune control of chronic HBV infection.

## Introduction

Hepatitis B virus (HBV) is a non-cytopathic hepadnavirus that chronically infects approximate 240 million people. Every year, HBV-associated end-stage liver disease results in about 1 million patient deaths (1). It is well known that the chance of clearing HBV infection is age-dependent and relies on the host immune system. Over 95% of adult patients who acquired HBV infections have spontaneous clearance, but 90% of neonates and 30% of children 1- to 5-year olds who acquired HBV infections fail to resolve the infection and develop chronic hepatitis B (CHB) (2, 3). Generally, the natural history of chronic HBV infection can be divided into different phases, including immune tolerance (IT), immune activation (IA), inactive carrier (IC), and reactivation (4, 5). During the IA phase, the host immune system, including both innate and adaptive immune responses, were activated to clear the virus from hepatocytes. A strong, diverse, and functional adaptive immune response is considered essential for HBV clearance. However, it has been clearly shown that deletion or exhaustion of adaptive immunity leads to HBV persistence in hepatocytes (6).

Liver inflammation accompanied by elevated levels of serum alanine aminotransferase (ALT) in CHB patients are usually observed and result from immune-mediated destruction of hepatic cells. Within the inflamed liver, there is an accumulation of lymphoid and myeloid cells, including T and B cells (7). These immune cells play critical roles in the clearance of HBV-infected hepatocytes but also are involved in immune-mediated liver damage and inflammation (8). Accumulating evidence indicates that certain chemokines in the liver are necessary for providing the appropriate environment for activation and expansion of naïve lymphocytes in response to hepatitis virus infection (9). Using a mouse model of HBV infection, it was found that chemokine C–X–C-chemokine ligand 13 (CXCL13), which is involved in hepatic B-lymphocyte trafficking and lymphoid architecture and development, is expressed in an age-dependent manner in mouse hepatic macrophages and plays an integral role in facilitating an effective immune response against HBV (10).

CXCL13 is a member of CXC subtype of chemokine superfamily and is also known as a B cell—attracting chemokine 1 or B-lymphocyte chemoattractant (11). By acting through its cognate receptor C–X–C chemokine receptor type 5 (CXCR5), CXCL13 is chemotactic for mature B cells and T follicular helper (Tfh) cells (12). Importantly, CXCL13 facilitates the co-migration of B cells and Tfh cells into B cell follicles and germinal centers (GCs), where high-affinity antibody-secreting memory B and plasma cells are generated (13). Aberrant circulating CXCL13 levels have been implicated in the pathogenesis of many chronic inflammatory diseases and correlated with clinical outcomes, including various infections and autoimmune disorders associated with dysregulated humoral responses (14–16).

Previous work from our group suggested that circulating Tfh cells might have a significant role in facilitating hepatitis B virus e antigen (HBeAg) seroconversion through interleukin-21 (IL-21) secretion in patients with chronic HBV infection (17). However, the association of CXCL13 expression with the pathogenesis of CHB and clinical outcome of antiviral therapy remains unclear. The present study was designed to examine CXCL13 expression and regulation in subpopulations of hepatic cells, as well as in the serum at different phases of CHB patients. In addition, we performed an in-depth prospective analysis of the kinetics of serum CXCL13 expression of CHB patients being treated with telbivudine with different clinical outcomes.

## Materials and Methods

### Study Subjects

Blood samples were obtained from 45 patients with chronic HBV infection who were recruited at Nanfang Hospital (Guangzhou, China) for a cross-sectional study. They were classified into three groups according to guidelines from the European Association for the Study of Liver Diseases: IA (*n* = 20); IT (*n* = 10), and IC (*n* = 15) (18). A total of 12 healthy controls (HCs) were enrolled for comparison. Demographic, biochemical, and virological features of the participants were listed in Table 1.

**Table 1:** **Clinical characteristics of the subjects**.

Group	IA	IT	IC	HC
No. of patients	20	10	15	12
Gender (M/F)	14/6	6/4	5/10	8/4
Age (years)	28.00 (21.00–41.80)	26.50 (3.80–34.70)	36.00 (22.60–54.40)	24.00 (21.30–34.40)
Hepatitis B virus (HBV) DNA (log_10_ copies/ml)	8.40 (5.94–9.39)	7.70 (5.55–8.70)	0.00^a^ (0.00–3.94)	ND
ALT (U/l)	145.00 (70.60–295.30)	22.00 (16.10–40.90)	18.00 (10.60–33.40)	15.00 (12.00–27.70)
AST (U/l)	90.50 (48.10–225.40)	24.50 (19.30–34.50)	25.00 (16.40–35.00)	22.00 (12.00–31.20)
HBsAg positive	20	10	12	0
Anti-HBs positive	0	0	1	6
Hepatitis B virus e antigen positive	20	5	5	0
Anti-Hbe positive	0	5	7	0
Anti-HBc positive	20	10	15	0
CXCL13 (pg/ml)	67.18 (46.77–180.51)	42.30 (23.92–85.69)	31.93 (17.46–98.42)	32.33 (13.69–49.90)

*Data were shown as median and 10–90% percentile*.

*^a^11 IC had undetectable HBV DNA*.

*ALT, alanine aminotransferase; AST, aspartate aminotransferase; HC, healthy control; IA, immune activation; IC, inactive carrier; IT, immune tolerance; ND, not determined*.

Fifty-five HBeAg-positive CHB subjects from Nanfang Hospital who had participated in two prospective clinical trials of telbivudine (600 mg/day, clinical trial numbers: NCT00962533 and CLDT600ACN07T) were also studied. According to treatment response at week 52, the participants were classified into a complete response (CR) group or a non-complete response (NCR) group (18). Subjects in the CR group had undergone ALT normalization and HBeAg seroconversion, and had achieved a serum HBV-DNA level of < 300 copies/ml by week 52; those in the NCR group had either a serum HBV-DNA level >300 copies/ml or were positive for HBeAg at week 52. All patients provided written documentation of informed consent. The study conforms to the ethical guidelines of the 1975 Declaration of Helsinki and was approved by the Ethical Committee of Nanfang Hospital.

### Serological Assays and HBV-DNA Assays

The presence of HBV surface antigen, HBeAg, anti-HBs, anti-HBe, and anti-HBc was determined using ARCHITECT i2000SR system (Abbott Ireland Diagnostics Division, Sligo, Ireland) (19). HBV DNA was measured using the COBAS TaqMan HBV Test (Roche Molecular Diagnostics, Pleasanton, CA, USA) (17), which has a detection limit of 12 IU/ml (1 IU/ml = 5.82 copies/ml).

### Enzyme-Linked Immunosorbent Assay (ELISA)

The concentration of CXCL13 was quantitated in duplicate wells using a commercial human CXCL13 ELISA kit (R&D Systems, Minneapolis, MN, USA) in accordance with the manufacturer’s instructions.

### PBMCs Isolation and Macrophage Differentiation

Peripheral blood mononuclear cells were separated on Ficoll-Histopaque (BD Biosciences, Shanghai, China) density gradients and routinely cryopreserved as previously described (17). The monocytes were isolated from PBMCs by adherence to plates coated with collagen-I (BD Biosciences, USA) at 37°C in RPMI 1640 containing 10% FBS (20). After incubation, the cells were washed by PBS for 5 min and then gently vibrated on a culture plate. The monocytes were then cultured for up to 9 days in the presence 10 ng/ml of GM-CSF (PerproTech, Rocky Hill, NJ, USA) in RPMI 1640 containing 10% FBS. This protocol resulted in more than 90% purity of the macrophages, as determined by flow cytometry analysis (FACS) using anti-CD14 APC-conjugated monoclonal antibody (BD Biosciences, USA). The differentiated macrophages were then stimulated for 6 h with 5 μg/ml of LPS or poly I:C (InvivoGen, San Diego, CA, USA).

### Primary Human Hepatocytes (PHHs) and Non-Parenchymal Cells Isolation and Incubation

Liver specimens (25–100 g) were obtained from fresh tumor resections or liver transplantation donors (HBV-positive, *n* = 6; HBV-negative, *n* = 6, patients’ information is in Table S1 in Supplementary Material). PHHs and non-parenchymal liver cells, including Kupffer cells (KCs), liver sinusoidal endothelial cells (LSECs), and hepatic stellate cells (HSCs), were prepared from a single human liver specimen according to a modified two-step perfusion technique, as described by our group recently (21). The study conforms to the ethical guidelines of the 1975 Declaration of Helsinki and was approved by the Institutional Review Board (ethics committee) of the medical faculty at the University of Duisburg-Essen. PHHs were seeded into collagen-I (BD Biosciences, USA) coated plates at a density of 1.25 to 2.5 × 10^5^ viable cells per cm^2^ by using DMEM/Ham’s F-12 medium (Biochrome, Berlin, Germany) supplemented with 10% FBS. KCs were seeded onto plastic culture plates at a density of 4 to 6 × 10^5^ cells per cm^2^ using DMEM supplemented with 10% FBS. LSECs were cultured in coated plates with Endothelial Growth Medium 2 (PromoCell, Heidelberg, Germany) containing provided supplements. HSCs were seeded into an uncoated plastic culture flask with Stellate Cell Medium (ScienCell, Carlsbad, CA, USA) supplemented with supplied 10% FBS, 1% stellate cell growth supplement. The purities of PHH (95.5 ± 1.7%), KC (94.5 ± 1.2%), LSEC (97.8 ± 1.1%), and HSC (97.1 ± 1.5%) could be reached as determined by immunofluorescence staining with specific markers (21). These cells were stimulated using the Human toll-like receptor (TLR) 1–9 Agonist Kit (Invivogen, Toulouse, France) at recommended concentrations for 6 h. Cells were then collected for RNA extraction and CXCL13 gene expression analysis by real-time RT-PCR was performed using commercially available primer set (Qiagen, Hilden, Germany) and normalized to β-actin as previously described (22).

### RNA Extraction, Reverse Transcription, and Quantitative Real-time PCR

Total cellular RNA was isolated and purified from PBMCs, stimulated macrophages or hepatic cells using the RNeasy Mini Kit (Qiagen, Hong Kong, China) according to the manufacturer’s instructions. The cDNA synthesis was performed with 200 ng total RNA using the Transcriptor First Strand cDNA Synthesis Kit (Roche, Mannheim, Germany). The expression of CXCL13 genes was determined by real-time RT-PCR, which was performed using the SYBR Green I Master Kit (Roche, Switzerland) on a LightCycler 480 (Roche Diagnostics, Switzerland), as described previously (23). The expression levels of each gene are presented as values normalized against 10^6^ copies of β-actin transcripts.

### Immunohistochemistry

Immunohistochemistry was performed on formalin-fixed and paraffin-embedded 4-μm sections of liver tissue. Sections were incubated overnight at 4°C with appropriate concentrations of primary antibodies, including rabbit anti-CD4, mouse anti-CD19, rabbit anti-CD38, mouse anti-CD68, and mouse anti-CXCR5 (Abcam, Cambridge, MA, USA) and goat anti-CXCL13 (R&D System, USA), and then incubated with the Dako Chemate Envision Kit (Dako, Glostrup, Denmark). The reaction was visualized by CheMate™ DAB plus chromogen (Dako, Denmark). The staining was captured by light microscopy using high-power microscopic fields (400×).

### Chemotaxis Assay

Chemotaxis was assessed in a two-chamber transwell cell migration system (5-μm pores, Corning, Shanghai, China) (24). PBMCs from CHB patients (1 × 10^6^) were purified as described above and were seeded in the upper well, and different concentrations of recombinant human CXCL13 (rh-CXCL13, PerproTech) or RPMI 1640 as controls were added to the bottom well as the chemoattractant. The cells were cultured for 2–6 h at 37°C and then collected and stained with CD19-APC, CD4-PE-Cy7, and CXCR5-PerCP-Cy5.5 (BioLegend, San Diego, CA, USA) The percentage of migrating cells was analyzed and calculated by a BD FACSCanto II flow cytometer.

### Statistical Analysis

Continuous data were expressed as medians (minimum–maximum). The Mann–Whitney *U* test, Wilcoxon’s signed-ranks test, and the chi-squared test were used for two-group comparisons. The Kruskal–Wallis *H* test was used when comparing more than three groups. Repeated measures analysis was used to compare changes in CXCL13 expression during treatment. The paired Student’s *t*-test was used to compare individual values at different times. Receiver-operating characteristic (ROC) curves were constructed to predict a CR to LdT treatment. All statistical analyses were based on two-tailed hypothesis tests with a significance level of *p* < 0.05.

## Results

### CXCL13 Expression in Liver Cells Is Regulated by TLR Agonists Stimulation

**Figure 1:**
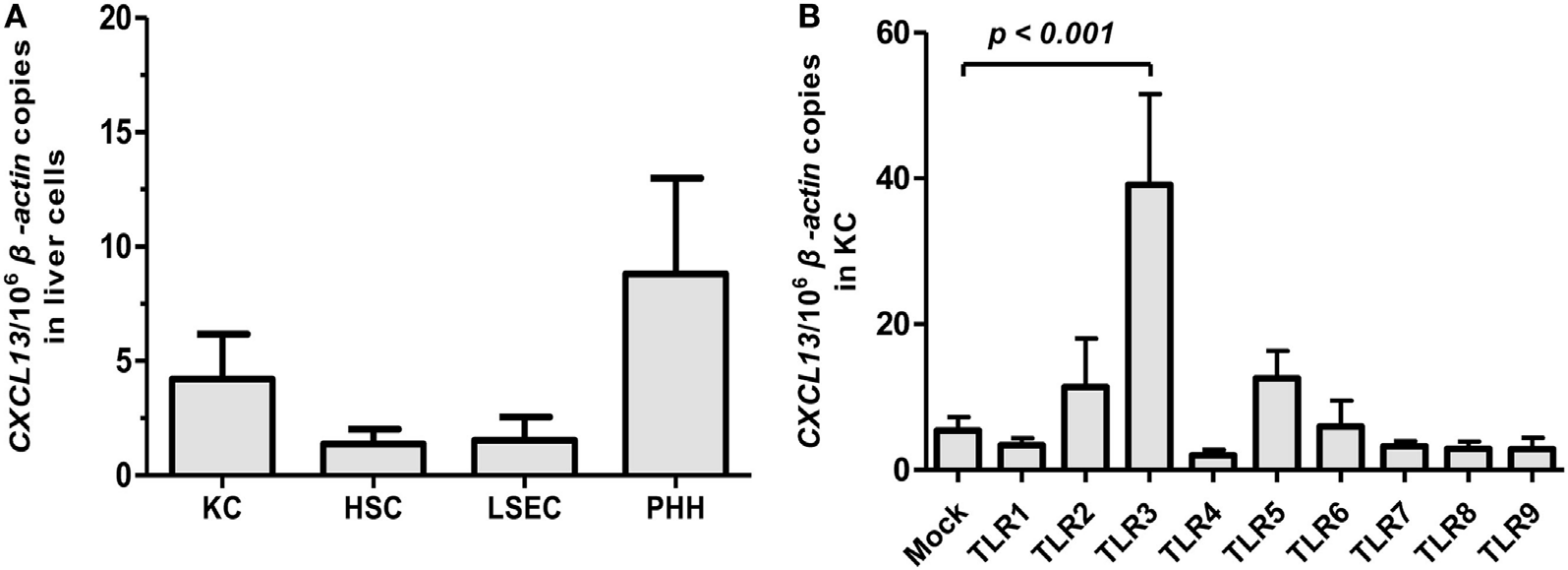
**CXCL13 expression in subpopulation of liver cells and its regulation by toll-like receptor (TLR) ligands**. **(A)** Comparison of CXCL13 mRNA expression in Kupffer cell (KC), hepatic stellate cell (HSC), liver sinusoidal endothelial cell (LSEC), and primary human hepatocyte (PHH). Different subpopulation of liver cells were isolated from non-hepatitis B virus-infected livers. The total RNA of these cells was extracted and the expression of CXCL13 mRNA was determined by real-time RT-PCR. **(B)** Comparison of CXCL13 mRNA expression in KC after stimulation with TLR1–9 ligands. Isolated KC were stimulated with different TLR1–9 ligands for 6 h and then harvested for RNA extraction. CXCL13 mRNA expression was determined by real-time RT-PCR. The stimulus and concentration used as below: TLR1/2 agonist: Pam3CSK4 (4 μg/ml), TLR2 agonist: HKLM (10^8^ cells/ml), TLR3 agonist: poly I:C (50 μg/ml), TLR4 agonist: LPS (30 μg/ml), TLR5 agonist: FLA (2 μg/ml), TLR6/2 agonist: FSL1 (1 μg/ml), TLR7 agonist: imiquimod (20 μg/ml), TLR8 agonist: ssRNA40 (10 μg/ml), TLR9 agonist: CpG (31.8 μg/ml). Data are representative with mean ± SEM, and determined by Mann–Whitney *U* test.

Previous studies in mouse model suggested that CXCL13 was predominantly expressed in liver macrophages (10). However, the expression of CXCL13 in human liver cells remains unknown. PHH and non-parenchymal liver cells composed of KC, LSEC, and HSC were isolated from fresh human liver specimens obtained from HBV-negative tumor resections of patients (*n* = 6). The CXCL13 mRNA expression in different liver cell subpopulations was examined by real-time RT-PCR. The basal expression of CXCL13 in KC, HSC, LSEC, and PHH isolated from non-HBV-infected livers was very low and had no significant difference (Figure 1A). Further, we investigated if CXCL13 expression was regulated by pathogen-associated molecular patterns; to do this we used various TLR1–9 agonists to stimulate the KC isolated from liver resections without HBV infection. We found that the CXCL13 mRNA expression could be induced by TLR3 agonist poly I:C for about 7.2-fold (*p* = 0.03), whereas other TLRs agonists had no significant effect (Figure 1B). As positive controls, interferon (IFN)-β induction was observed in poly I:C stimulation and all TLR ligands, except TLR9 ligand CPG, were able to induce IL-6 expression at different levels in KC (Figures S1A,B in Supplementary Material). Consistently, by using *in vitro* differentiated macrophages from monocytes (Figure S2A in Supplementary Material), poly I:C were able to induce CXCL13 expression at mRNA (Figure S2B in Supplementary Material) and protein levels (Figure S2C in Supplementary Material).

### KC Isolated from HBV-Positive Liver Tissues Express Higher Levels of CXCL13 in Response to TLR3 Agonist Poly I:C Compared to KC with HBV-Negative Origin

**Figure 2:**
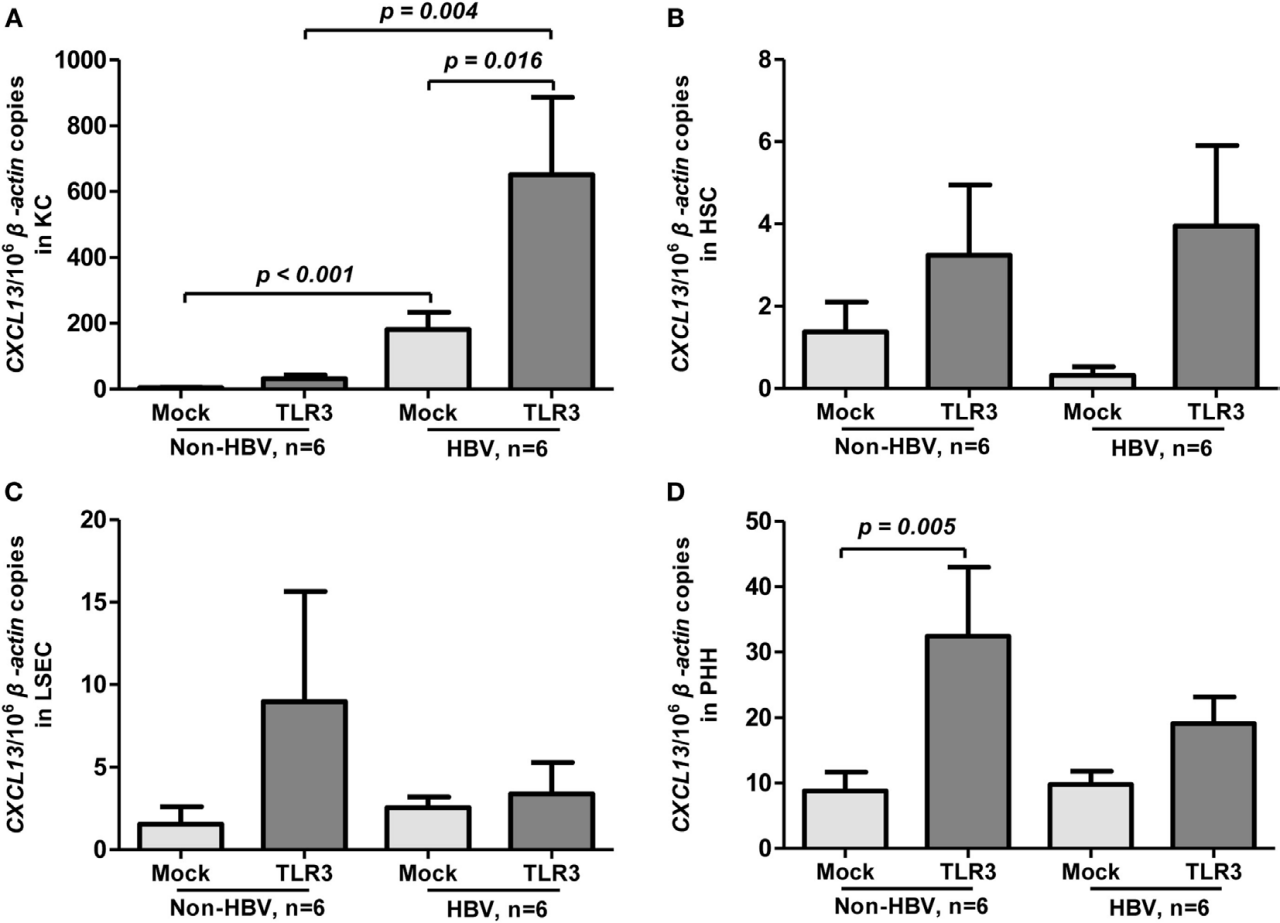
**Toll-like receptor (TLR) 3 agonist-induced expression of CXCL13 in liver cells from hepatitis B virus (HBV)-positive and non-HBV-infected patients**. Comparison of the basal and TLR3 agonist-induced expression of CXCL13 in Kupffer cell (KC) **(A)**, hepatic stellate cell (HSC) **(B)**, liver sinusoidal endothelial cell (LSEC) **(C)**, and primary human hepatocyte (PHH) **(D)**. Different subpopulation of liver cells were isolated from either Non-HBV or HBV-positive subjects and stimulated with or without TLR3 agonist for 6 h. Total RNA was extracted and then the CXCL13 mRNA expression to β-actin were determined by real-time RT-PCR. Data are representative with mean ± SEM, and determined by Mann–Whitney *U* test and Wilcoxon’s signed-ranks test.

To assess whether HBV infection could influence CXCL13 expression, we compared the basal and TLR3 agonist-induced expression of CXCL13 in liver cell subpopulations between HBV-positive and HBV-negative patients (*n* = 6 for each group). Interestingly, the KC isolated from HBV-positive patients had much higher level of CXCL13 expression than non-HBV patients (about 45-fold, *p* < 0.001, Figure 2A). Upon poly I:C stimulation, the induced CXCL13 expression in KC isolated from HBV-positive patients was also markedly higher than in non-HBV patients (Figure 2A, *p* = 0.004). However, the basal expression of CXCL13 in other cells including HSC (Figure 2B), LSEC (Figure 2C), and PHH (Figure 2D) had no significant difference among these two groups. Moreover, poly I:C stimulation had very weak effect on inducing CXCL13 expression in these cells, except PHH (*p* = 0.005).

### Serum CXCL13 Concentrations Is Increased in the Immune-Activation Phase of Chronic HBV Infection and Negatively Correlated with HBV-DNA Levels

**Figure 3:**
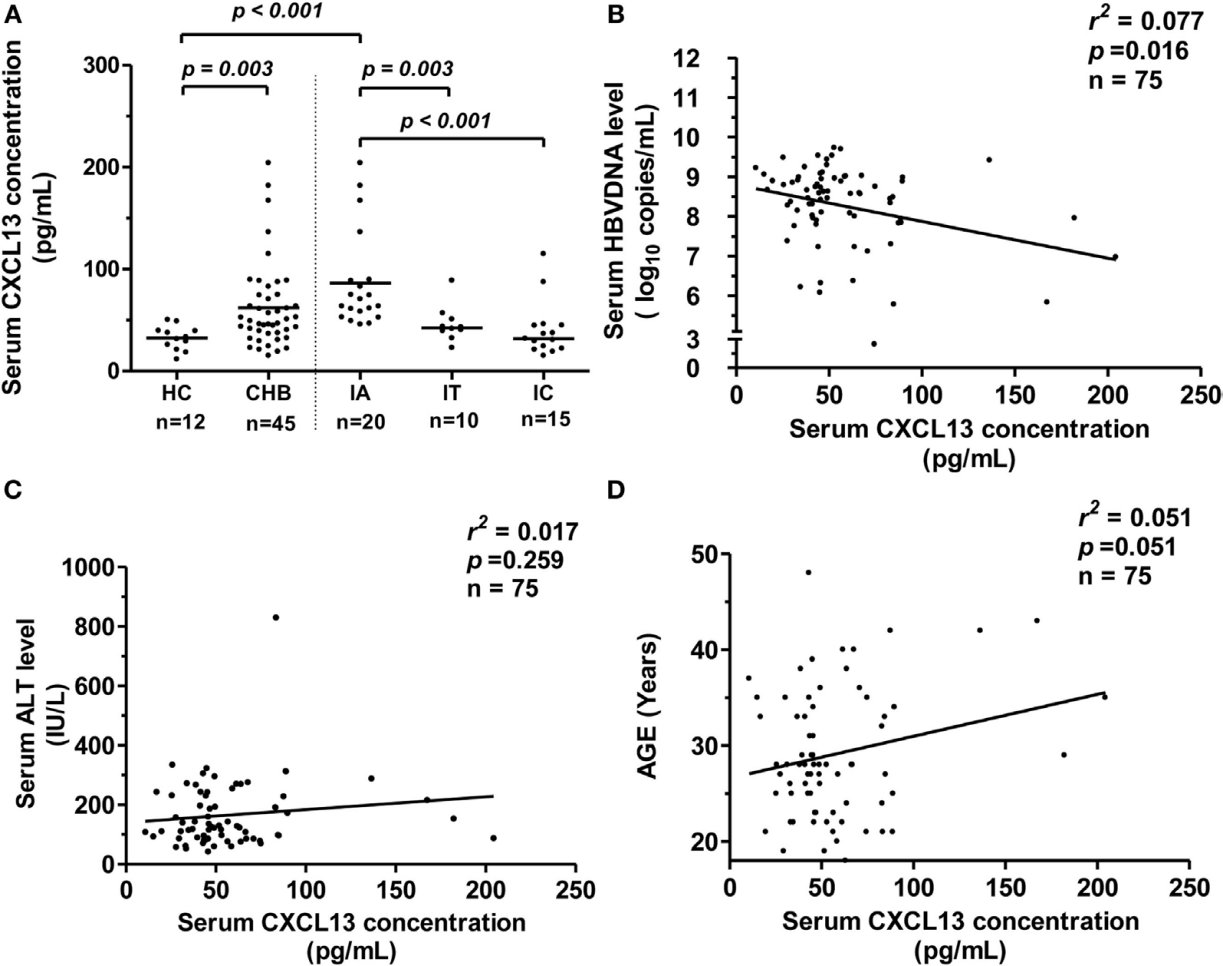
**CXCL13 expression in serum samples from chronic hepatitis B (CHB) patients and its correlation with viral parameters**. **(A)** Comparison of serum CXCL13 concentration in healthy control (HC) and CHB patients with chronic hepatitis B virus (HBV) infection [divided into immune activation (IA), IT, and IC groups]. Serum CXCL13 concentrations were measured by specific enzyme-linked immunosorbent assay kit. Data are representative with median, and determined by Mann–Whitney *U* test. **(B)** The correlation between serum CXCL13 concentrations and HBV DNA (log_10_ copies/ml) in IA patients, including the baseline of CHB patients received telbivudine therapy. **(C)** The correlation between serum CXCL13 concentrations and alanine aminotransferase (ALT) levels in IA patients. **(D)** The correlation between serum CXCL13 concentrations and the age in IA patients.

To elucidate whether serum CXCL13 levels in patients could also be affected by HBV infection, we examined the concentration of CXCL13 by ELISA in CHB patients. We saw that serum CXCL13 was significantly higher in CHB patients than in HCs (Figure 3A, *p* = 0.003). The CHB patients were further divided into three groups (IA, IT, and IC) depending on ALT levels and viral parameters. The median serum CXCL13 concentrations in the IA groups were higher compared with the IT, IC, and HC groups, and there was no significant difference among the IT, IC, and HC groups (Figure 3A). Serum CXCL13 concentrations were negatively and weakly correlated with serum HBV-DNA levels in CHB patients (Figure 3B, *r*^2^ = 0.077, *p* = 0.016) but were uncorrelated with ALT levels (Figure 3C, *p* = 0.259). Consistent with previous data in human infant and adult samples (10), there was a weak positive correlation between serum CXCL13 concentration and age (Figure 3D, *r*^2^ = 0.051, *p* = 0.051).

### Dynamic Change of Serum CXCL13 Concentrations under Telbivudine Therapy Correlates with Treatment Outcome

**Figure 4:**
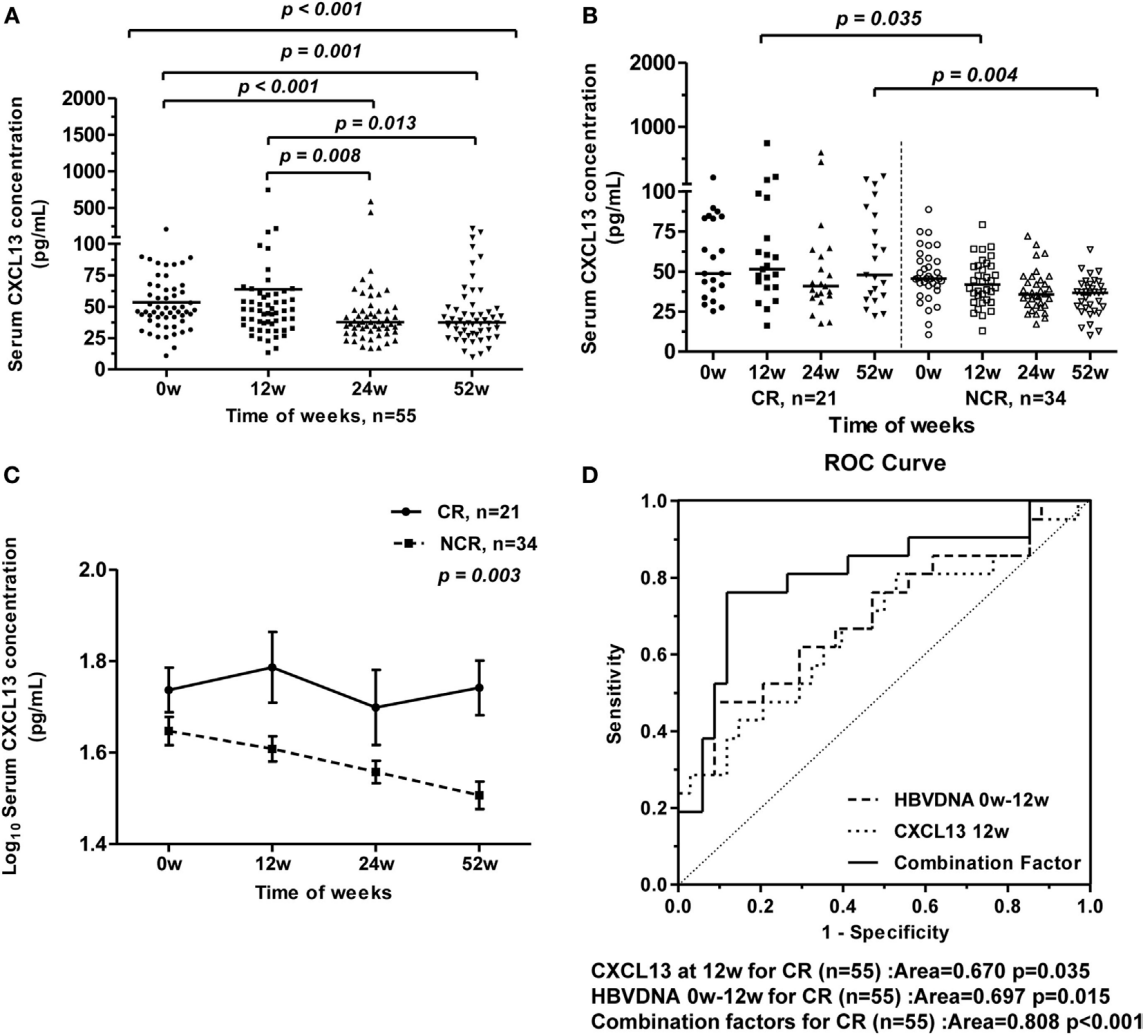
**Longitudinal analysis of serum CXCL13 concentrations of chronic hepatitis B (CHB) patients during telbivudine therapy**. **(A)** Comparison of serum CXCL13 concentrations at baseline and at 12, 24, and 52 weeks after starting telbivudine therapy in all 55 hepatitis B virus e antigen-positive CHB patients. Data are representative with median, and determined by Mann–Whitney *U* test and Kruskal–Wallis H test. **(B)** Comparison of serum CXCL13 concentrations between complete response (CR) group and non-complete response (NCR) group at indicated time points. Data are representative with median, and determined by Mann–Whitney *U* test. **(C)** Repeated measures analysis of temporal dynamics of serum CXCL13 concentrations between CR group and NCR group at indicated time points. The paired Student’s *t*-test was used to compare individual values at different time points. **(D)** Receiver-operating characteristic (ROC) curves for prediction of CR after telbivudine therapy, including CXCL13 concentrations at week 12, log_10_ HBV DNA copies/ml of week 0 − week 12, and combination factors above.

To assess changes of serum CXCL13 concentrations in HBV-infected patients during nucleoside analog therapy, we studied 55 HBeAg-positive CHB patients who received 52 weeks of telbivudine treatment. During this antiviral treatment, the serum CXCL13 concentrations in all patients gradually decreased from 0 to 52 weeks of therapy, while the serum CXCL13 concentrations at baseline and 12 weeks were higher than at 24 and 52 weeks (Figure 4A). These patients were further divided into CR and NCR group as described in Section “Materials and Methods” (19). The features (age and sex, as well as serum ALT, AST, TBIL, HBV-DNA, and CXCL13 levels) of the CR group and NCR group from baseline to 52 weeks during therapy were compared (Table 2). Mean ALT levels of the CR group decreased to normal at week 12, and were much lower than in the NCR group at 12 weeks (*p* = 0.042). Serum HBV-DNA levels were lower in CR group than NCR group at 12, 24, and 52 weeks (*p* < 0.005). Furthermore, the serum CXCL13 was higher in the CR than the NCR group at 12 and 52 weeks (Figure 4B). Repeated measures analysis showed that serum CXCL13 concentration decreased in the NCR group during therapy but in the CR group it remained largely unchanged (Figure 4C). Finally, ROC curves were generated to assess the usefulness of 12-week CXCL13 concentrations, 0-week log_10_ HBV DNA− 12-week log_10_ HBV DNA, and combination factors between them to predict a CR at 52 weeks (Figure 4D). The combination factor was calculated from logistic regression analysis between CXCL13 concentrations and value of 0-week log_10_ HBV DNA− 12-week log_10_ HBV DNA. The optimal cut-off value for the 12-week CXCL13 concentration was 58.1 pg/ml. This indicated that sensitivity for detection of a CR was 42.9% with a specificity of 85.3%. The optimal cut-off value for log_10_ HBV DNA was 5.2, which provided sensitivity for detection of a CR of 47.6% and a specificity of 91.2%. The optimal cut-off value for combination factors was 0.41. This provided detection sensitivity for a CR of 76.2% and a specificity of 88.2%.

**Table 2:** **Univariate analysis of routine markers possibly associated with hepatitis B virus e antigen seroconversion**.

Variable	TW	Complete response	Non-complete response	χ^2^/*t*/*Z*	*p*
No. of patients		21	34		
Male (*n*, %)^a^		16 (76.2%)	28 (82.6%)	0.308	0.579
Age (years)^b^		27.00 (19.40–37.40)	28.00 (21.00–35.50)	0.110	0.913
ALT (U/l)^b^	0	174.00 (85.40–313.80)	115.50 (59.00–301.00)	1.841	0.071
	12	28.00 (16.00–85.20)	50.00 (17.00–125.00)	−2.088	0.042^#^
	24	20.00 (12.40–28.80)	24.00 (13.50–63.00)	−1.944	0.057
	52	20.00 (12.20–35.00)	19.00 (11.50–42.00)	−0.654	0.516
AST (U/l)^b^	0	105.00 (48.20–152.40)	86.00 (43.00–179.50)	0.695	0.490
	12	31.00 (21.40–46.60)	38.00 (25.00–58.50)	−1.803	0.077
	24	25.00 (19.20–36.40)	26.00 (20.50–39.50)	−1.324	0.191
	52	30.00 (18.60–76.40)	30.50 (19.50–51.50)	1.218	0.229
TBIL (μmol/ml)^b^	0	15.20 (8.72–25.26)	13.60 (8.45–22.10)	0.471	0.640
	12	11.30 (7.34–19.56)	13.20 (8.65–20.60)	−1.298	0.200
	24	12.10 (6.56–24.74)	13.35 (7.60–23.65)	−1.174	0.246
	52	13.00 (7.94–21.94)	12.10 (8.15–22.45)	−0.369	0.714
Hepatitis B virus DNA (log_10_ copies/ml)^b^	0	8.37 (6.49–9.41)	8.66 (7.11–9.53)	−0.699	0.487
	12	3.27 (2.14–4.70)	4.14 (3.11–5.55)	−4.430	0.000^#^
	24	2.34 (1.82–3.73)	3.36 (2.29–5.35)	−4.212	0.000^#^
	52	1.84 (1.81–2.46)	2.19 (1.81–5.97)	−3.306	0.002^#^
CXCL13 (pg/ml)^c^	0	48.77 (27.87–89.09)	45.51 (26.67–70.97)	−1.109	0.268
	12	51.42 (27.19–203.65)	41.80 (24.55–63.96)	−2.105	0.035^#^
	24	40.90 (19.14–374.20)	35.61 (23.37–60.83)	−1.516	0.130
	52	47.88 (24.10–160.68)	36.77 (15.71–49.50)	−2.876	0.004^#^

*Data were shown as median and 10–90% percentile*.

*^a^Chi-square test*.

*^b^Independent samples test*.

*^c^Mann–Whitney test*.

*TW, treatment week; IL, interleukin; TBIL, total bilirubin; ULN, upper limit of normal*.

*^#^p < 0.05*.

### CXCL13 Is Involved in the Formation of Ectopic GC in the Liver Tissue of CHB Patients

**Figure 5:**
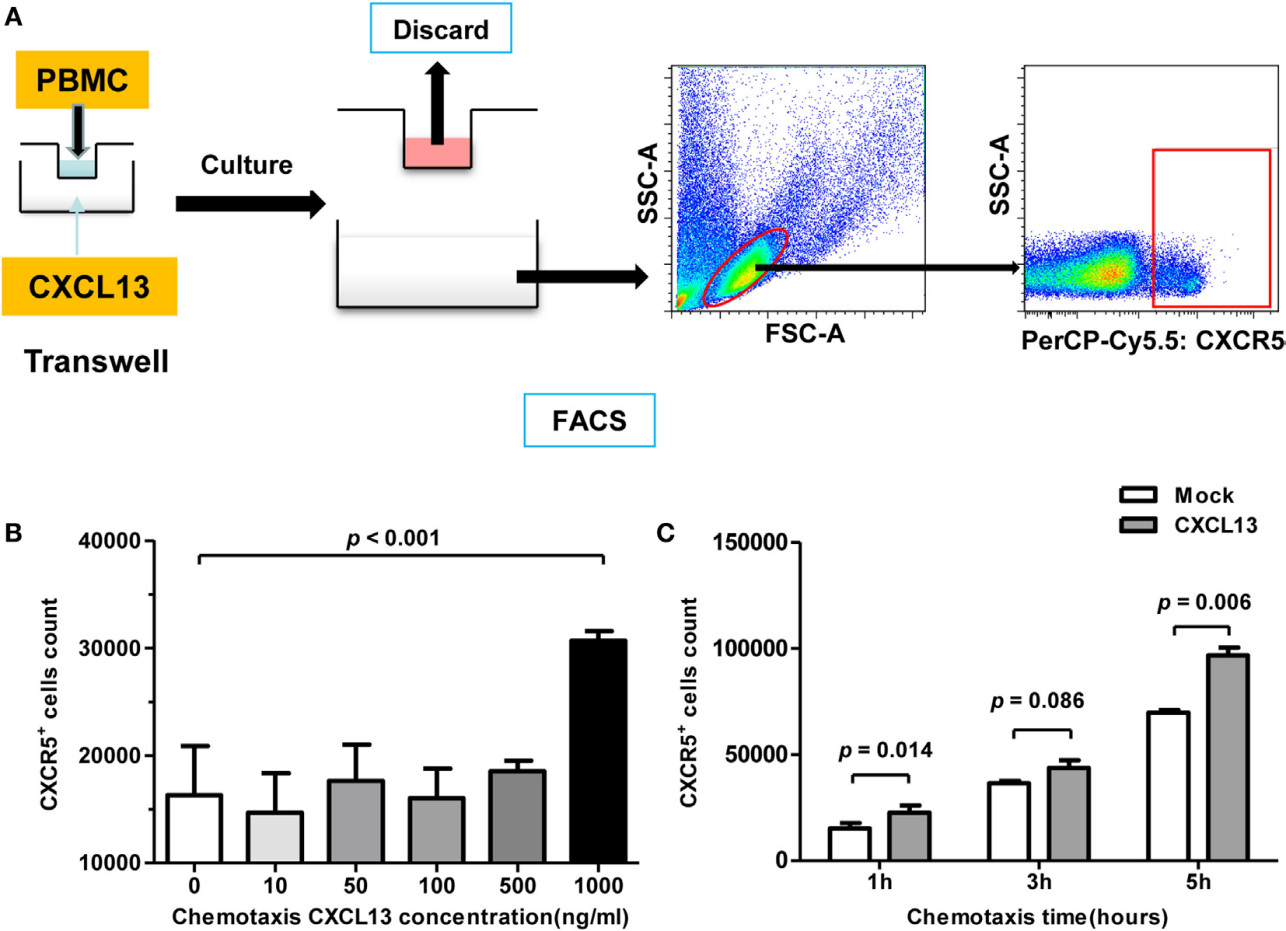
**CXCL13-mediated chemotaxis effect on CXCR5^+^ cells *in vitro***. **(A)** Schematic diagram of chemotaxis assay. A 1 × 10^6^ fresh-isolated peripheral blood mononuclear cells were added into the upper chamber, and different concentration of rh-CXCL13 or median alone was added into lower chamber. After 2–6 h, the cells in the lower chamber were harvested and analyzed. **(B)** Comparison of the numbers of CXCR5^+^ cell counting in chronic hepatitis B (CHB) patients with different CXCL13 concentration in Transwell assay. Data are representative with mean ± SEM, and determined by Mann–Whitney *U* test. **(C)** Comparison of the numbers of CXCR5^+^ cell counting in CHB patients with different incubation time in Transwell assay. Data are representative with mean ± SEM, and determined by paired Student’s *t*-test.

CXCL13 is known to dictate homing and motility of CXCR5^+^ lymphocytes, we examined the chemotaxis using CHB patient PBMCs by Transwell test (Figure 5A). With a concentration of 1 μg/ml CXCL13 in the bottom, the cell counting of CXCR5^+^ lymphocytes that moved through the membrane increased significantly (Figure 5B). In addition, with the response time extended, an increasing number of CXCR5^+^ lymphocytes moved through the Transwell filter membrane, and univariate *post hoc* analysis indicated that CXCL13 chemotaxis appeared to be time dependent (Figure 5C).

**Figure 6:**
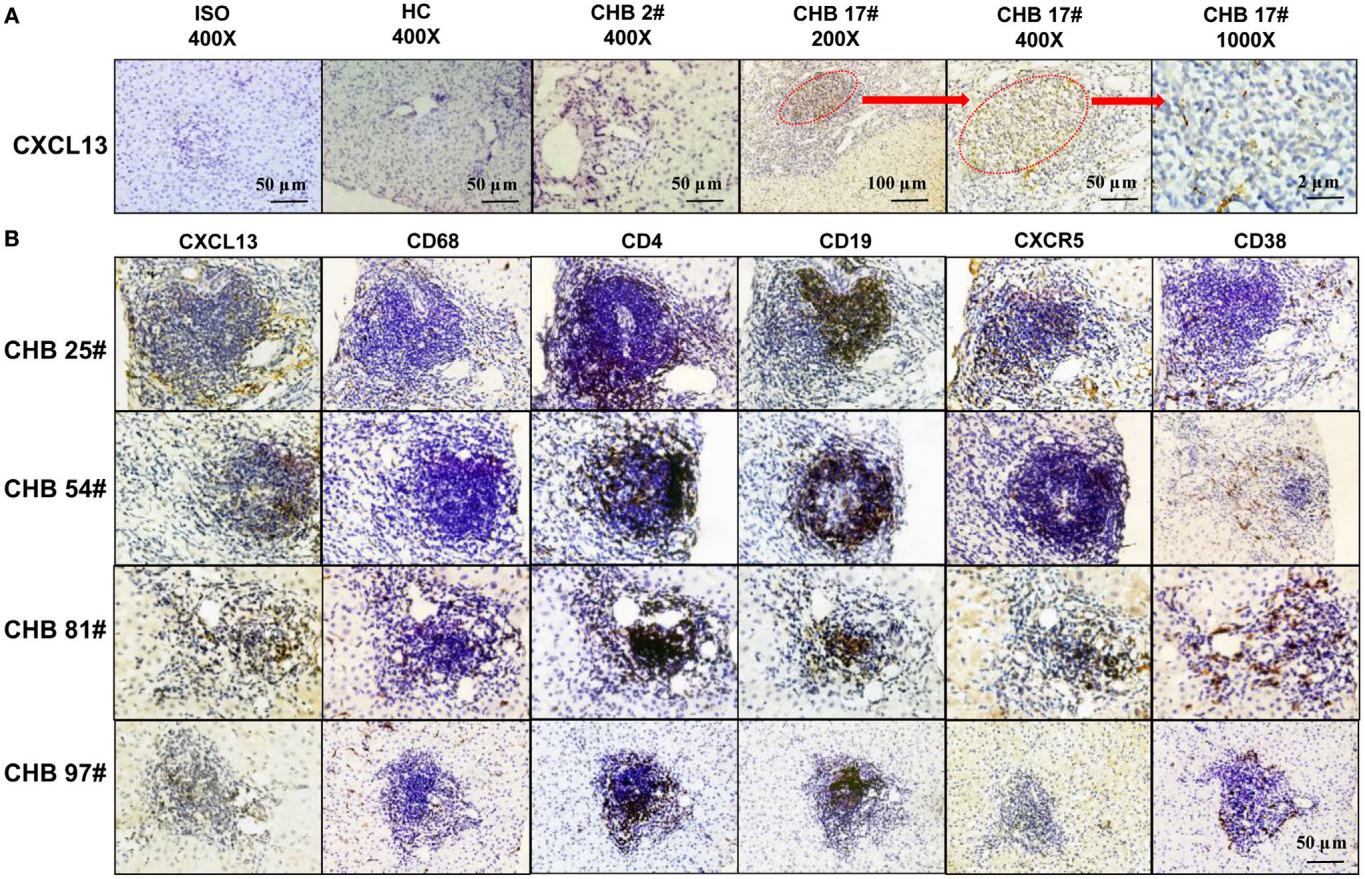
***In situ* expression of CXCL13 in the ectopic germinal center (GC) of liver tissue from chronic hepatitis B (CHB) patients**. **(A)** Immunohistochemical staining of CXCL13 expression in liver tissue samples with or without ectopic GC from CHB patients and healthy control (HC). Representative slides were shown with different magnifications as indicated. **(B)** Immunohistochemical staining of CXCL13^+^, CD4^+^, CD19^+^, CXCR5^+^, CD38^+^, and CD68^+^ cells in serial sections of liver tissue samples with ectopic GC from four representative CHB patients. Co-localization of CXCL13 expression was observed with CD68^+^ KCs, CXCR5^+^ CD4^+^ T cells, and CXCR5^+^CD19^+^ B cells in the ectopic GC (magnification ×400, plotting scale equal to 50 μm).

In order to verify the co-localization of CXCL13 and CXCR5^+^ in cells, we performed immunohistochemical staining of serial sections from liver samples. The staining of CXCL13 was very low in the liver tissue of most CHB patients and all HC group patients. However, there was positive CXCL13 staining in ectopic GCs of CHB liver biopsy samples (Figure 6A). Interestingly, co-localization of CXCL13 with CD68^+^ KCs could be seen in serial sections of the liver biopsies, which reflected an accumulation of CXCR5^+^ CD4^+^ T cells, and CXCR5^+^CD19^+^ B cells in the ectopic GCs in CHB liver, and CD38^+^ cells accumulation surrounding the GCs (Figure 6B). These results suggest that CXCL13 was involved in the recruitment of CXCR5^+^ lymphocytes in the liver of CHB patients.

## Discussion

In the present study, we examined the CXCL13 expression in different hepatic cells from CHB patients and found that CXCL13 was highly expressed and induced by poly I:C stimulation in KC isolated from HBV-positive livers. Serum CXCL13 concentrations were consistently increased and negatively correlated with HBV-DNA titers in immune-active CHB patients. Moreover, serum CXCL13 in CR patients was found to be significantly higher than in NCR patients beginning at 12 weeks of telbivudine treatment. This suggests a role for CXCL13 in predicting CR to antiviral therapy. The findings presented here provide further evidence to support CXCL13 as an important chemokine for immune control of HBV infection.

Chronic hepatitis B has been associated with chronic inflammation mediated by immune cells, inflammatory cytokines, and chemokines (25). KCs are tissue-resident macrophages and are crucial cellular components of the intrahepatic innate immune system (26). Based on their localization, KCs are likely to interact with HBV. However, the role of KCs in inducing immunity toward HBV is poorly understood (27). As we found with results from the HBV transgenic mice model, we confirmed that KCs from HBV-positive patients had higher level of CXCL13 expression. It is likely that HBV infection could stimulate CXCL13 expression, as we observed that only TLR3 ligand poly I:C stimulation induced robust CXCL13 production. Although it is unlikely that HBV replicates in KC, activation of KC by HBV, and its proteins has been demonstrated. Hösel et al. (28) showed that HBV particles and HBsAg induce IL-1β, IL-6, CXCL8, and TNF-α production in human KC *via* NF-κB activation. Boltjes et al. (29) demonstrated that a direct interaction between HBsAg and KC resulted in HBsAg uptake, induction of cytokine production, and the induction of IFN-γ production by NK cells. Alternatively, phagocytosis of HBV itself or -infected hepatocytes by KC may allow intracellular TLR3 exposure to viral RNA (30, 31).

Additionally, CXCL13 expression in KC may be induced by the ongoing hepatic inflammation, which maintains the pathologic process in the tissue by attracting additional lymphocytes and leading to chronic damage. Under chronic HBV infection, serum CXCL13 levels were found to be elevated in CHB patients compared with HCs. The highest levels occurred in immune-active patients. It can be interpreted that the high serum levels of CXCL13 may be a consequence of high local production. In our study, CXCL13 was highly expressed in the ectopic GC of CHB liver, co-localized with KC, Tfh cells, and B cells, indicating that KC-expressed CXCL13 served as a critical regulator for the recruitment of CXCR5^+^ lymphocytes in CHB patients. Previously, the immunohistochemical findings in liver biopsies suggested a major role for B-cells in the pathogenesis of CHB, which appeared to be driven by increased CD20^+^ B-cells (32). In addition, we also completed a comprehensive gene-chip analysis of intrahepatic gene expression in patients at different phases of HBV infection. Consistently, CHB patients had significantly elevated expression of CXCL13 in liver tissues compared to the IT and IC groups (data not shown). However, no direct correlation between CXCL13 serum levels and ALT was detected in our study. Although these findings may indicate a lack of a direct effect of CXCL13 on liver inflammation, they do highlight the *in situ* relation between CXCL13 and CXCR5^+^ lymphocytes.

The role of CXCL13 expression has already investigated in other types of chronic hepatitis (15, 16). Our group reported before that the levels of serum CXCL13 were significantly higher in primary biliary cirrhosis patients compared to HC. And there was a significantly higher mRNA expression of intrahepatic CXCL13 relative to HC and it suggested that CXCL13 promoted intrahepatic CXCR5^+^ lymphocyte homing and aberrant B cell immune responses in PBC (15). We also compared the serum CXCL13 levels and liver CXCL13 expression in PBC and CHB patients. It was noted that the levels of serum CXCL13 were significantly higher in PBC than CHB. *In situ* staining revealed significantly higher expression of CXCL13 within the portal tracts in PBC than CHB (Figures S3A,B in Supplementary Material). Additionally, serum CXCL13 levels were found to be elevated in chronic hepatitis C virus infection compared with HC and expression of CXCL13 protein was evident in inflammatory cells within portal tracts and strongly correlated with CD20^+^ B-cell numbers (16, 33). Thus, further studies are necessary to focus and identify the relationships and contributions of CXCL13 to patients with chronic hepatitis at different stages of inflammatory index of liver histology.

To establish whether CXCL13 changes contributed to the success of antiviral therapy, serum levels of this chemokine were monitored serially throughout the course of telbivudine treatment. CXCL13 levels did not change after CR to antiviral therapy. On the contrary, CXCL13 significantly declined in NCR group during the treatment. ROC curves of the serum CXCL13 concentrations at 12 weeks were generated to predict a CR at 52 weeks, but the sensitivity and specificity for detection of a CR were not as satisfactory as HBV-DNA levels. Of note, a negative correlation between CXCL13 serum levels and circulating viral load was noted. Although the combination of weeks 0–12, HBV-DNA level and serum CXCL13 concentration showed an optimal index to predict the CR, the predication value of the ROC curve may hard to be applied for clinical guidance due to the limited sample size of the patients in this study. In addition, as CXCL13 expression might be induced by the ongoing hepatic inflammation, the amelioration of liver inflammation with normalization of ALT levels explanted the decrease of CXCL13 expression during therapy. However, the higher level of CXCL13 in CR group referred to the activation of local anti-HBV immune response to contribute to viral control. Related results in our group showed the similar tendency of these immune factors during the antiviral treatment (17, 19). It was showed serum IL-21 concentration and the frequency of circulating Tfh cells were significantly higher in CR group compared with NCR group. The significant difference between CR and NCR was observed at treatment 12 weeks, the time when an increase in the IL-21-secreting Tfh cells that are associated with HBeAg seroconversion can be detected (17, 19). It could be assumed that Tfh cells and B cells would be attracted by CXCL13 and migrate to the liver, where the Tfh cells would secrete IL-21 and promote B cell maturation and production of antibodies.

In conclusion, we have observed that patients with chronic HBV infection have increased serum levels of CXCL13, possibly as the result of augmented production in the liver by KC. In CHB patients, CXCL13 contributes to lymphocyte migration to the liver by creating local microenvironments supportive of focal B-cell aggregations that have structural features remarkably similar to ectopic lymphoid follicles. Higher CXCL13 production seems to be involved in viral control during antiviral treatment, which suggests the possible use of serum CXCL13 monitoring to follow the treatment response to antiviral therapy and the prognosis. Future studies are also needed to determine whether TLR agonists that activate CXCL13 expression represent potential therapeutic agents for HBV infections.

## Author Contributions

XZ and JH conceived and designed the study. CL, XH, MW, RB, JG, YL, and BL performed the experiments and analyzed the data. XZ and CL wrote the manuscript with additional input and suggestions from JS, JP, and ML. All authors reviewed and approved the manuscript.

## Conflict of Interest Statement

The authors have nothing to disclose with respect to funding and no conflicts of interest with respect to this manuscript.

## Abbreviations

ALT, alanine aminotransferase; CHB, chronic hepatitis B; CR, complete response; CXCL13, C–X–C-chemokine ligand 13; CXCR5, C–X–C chemokine receptor type 5; ELISA, enzyme-linked immunosorbent assay; FDC, follicular dendritic cell; GCs, germinal centers; HBeAg, hepatitis B virus e antigen; HBV, hepatitis B virus; HC, healthy control; HCV, hepatitis C virus; HSC, hepatic stellate cell; IA, immune activation; IC, inactive carrier; IFN, interferon; IL-21, interleukin-21; IT, immune tolerance; KC, Kupffer cell; LSEC, liver sinusoidal endothelial cell; NCR, non-complete response; NPCs, non-parenchymal cells; PBMC, peripheral blood mononuclear cell; PHH, primary human hepatocyte; TBIL, total bilirubin; TFH, follicular T helper cells; TLR, toll-like receptor.
