# Supplementary figures and images for: Elevated Expression of Chemokine CXCL13 in Chronic Hepatitis B Patients Links to Immune Control during Antiviral Therapy
Source: Front Immunol. 2017 Mar 23;8:323. doi: 10.3389/fimmu.2017.00323 (PMC5362616; doi:10.3389/fimmu.2017.00323)

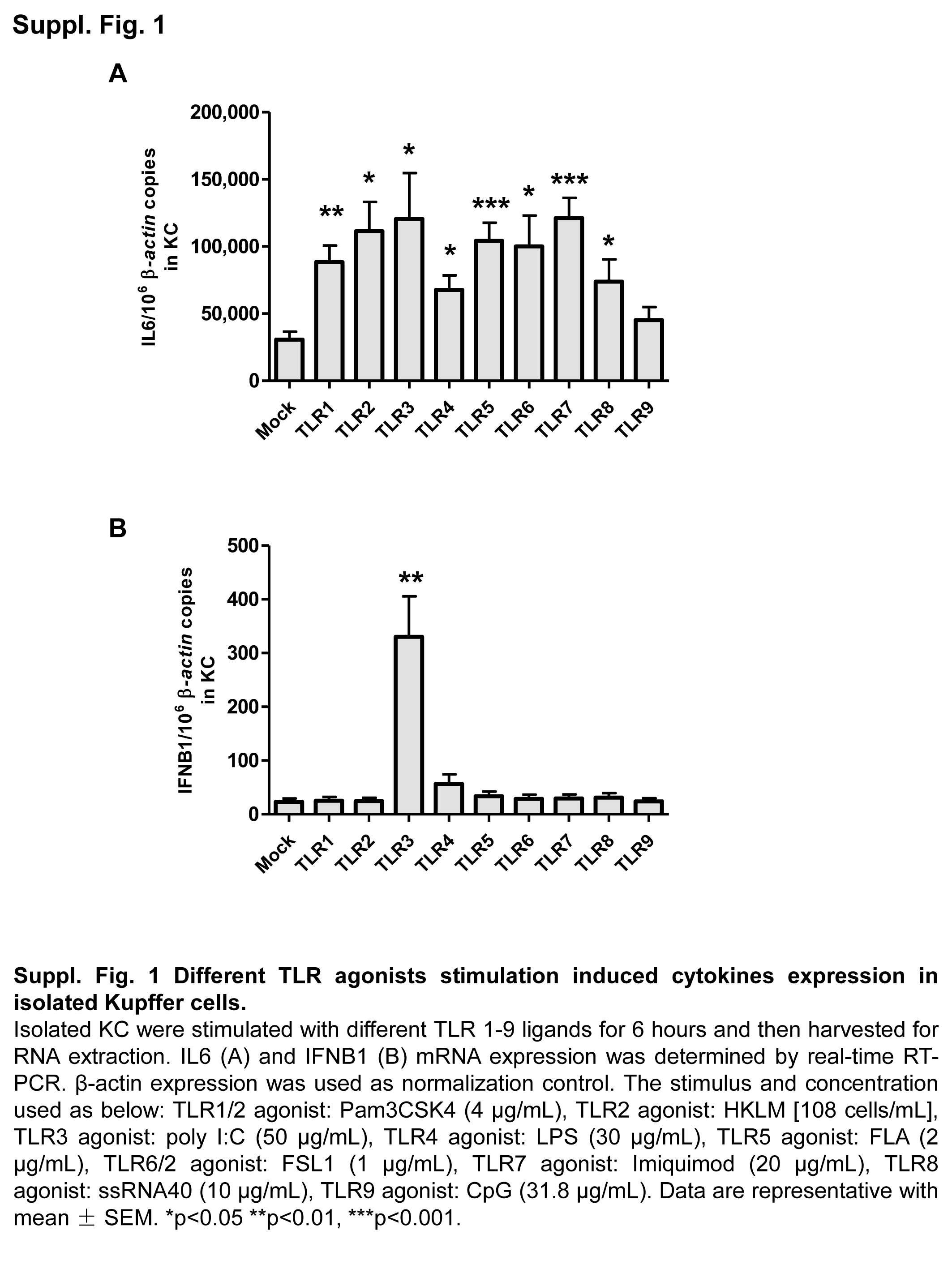

Supplement: Supplementary file 1 [file image_1.tif]

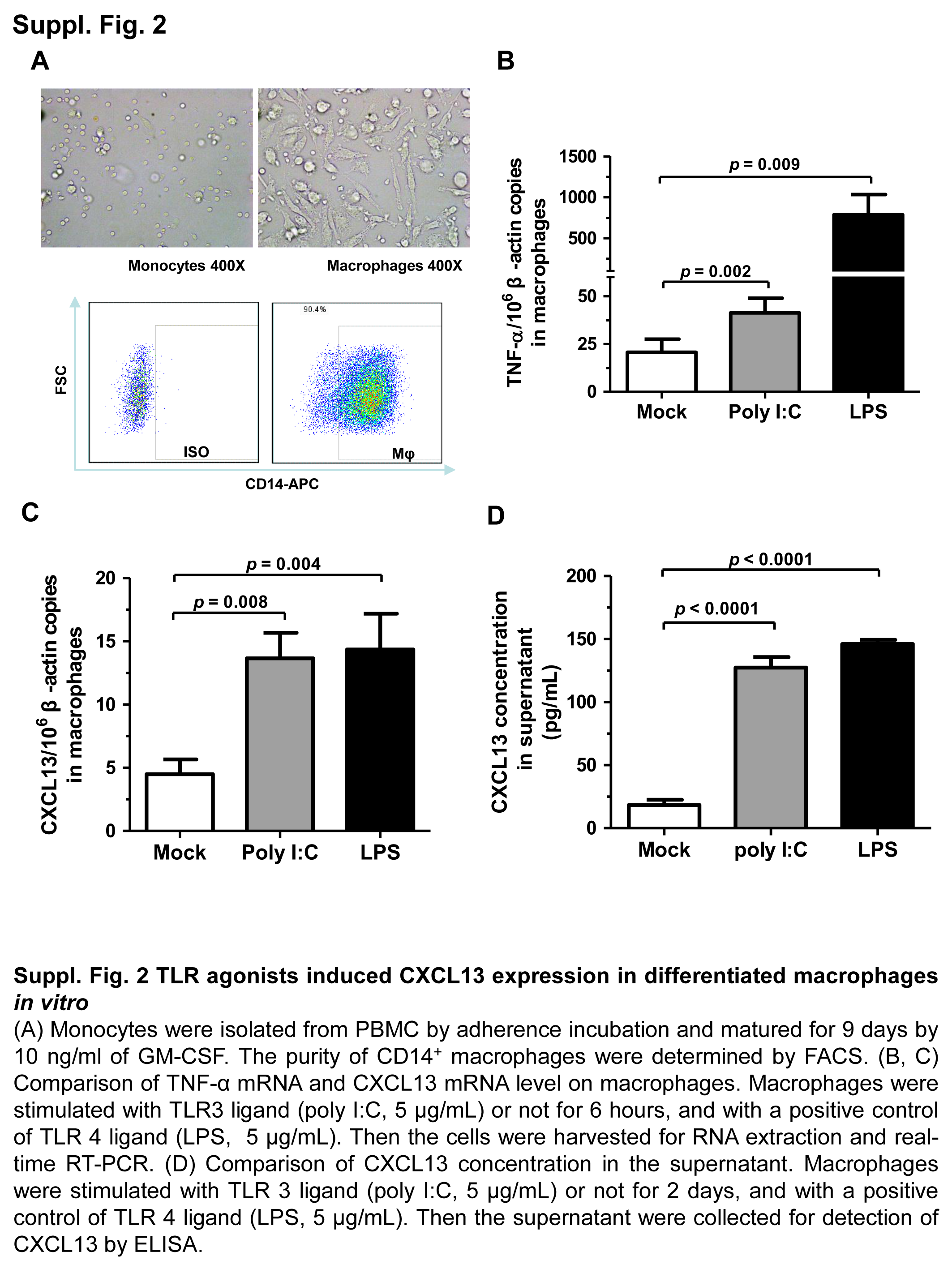

Supplement: Supplementary file 2 [file image_2.tif]

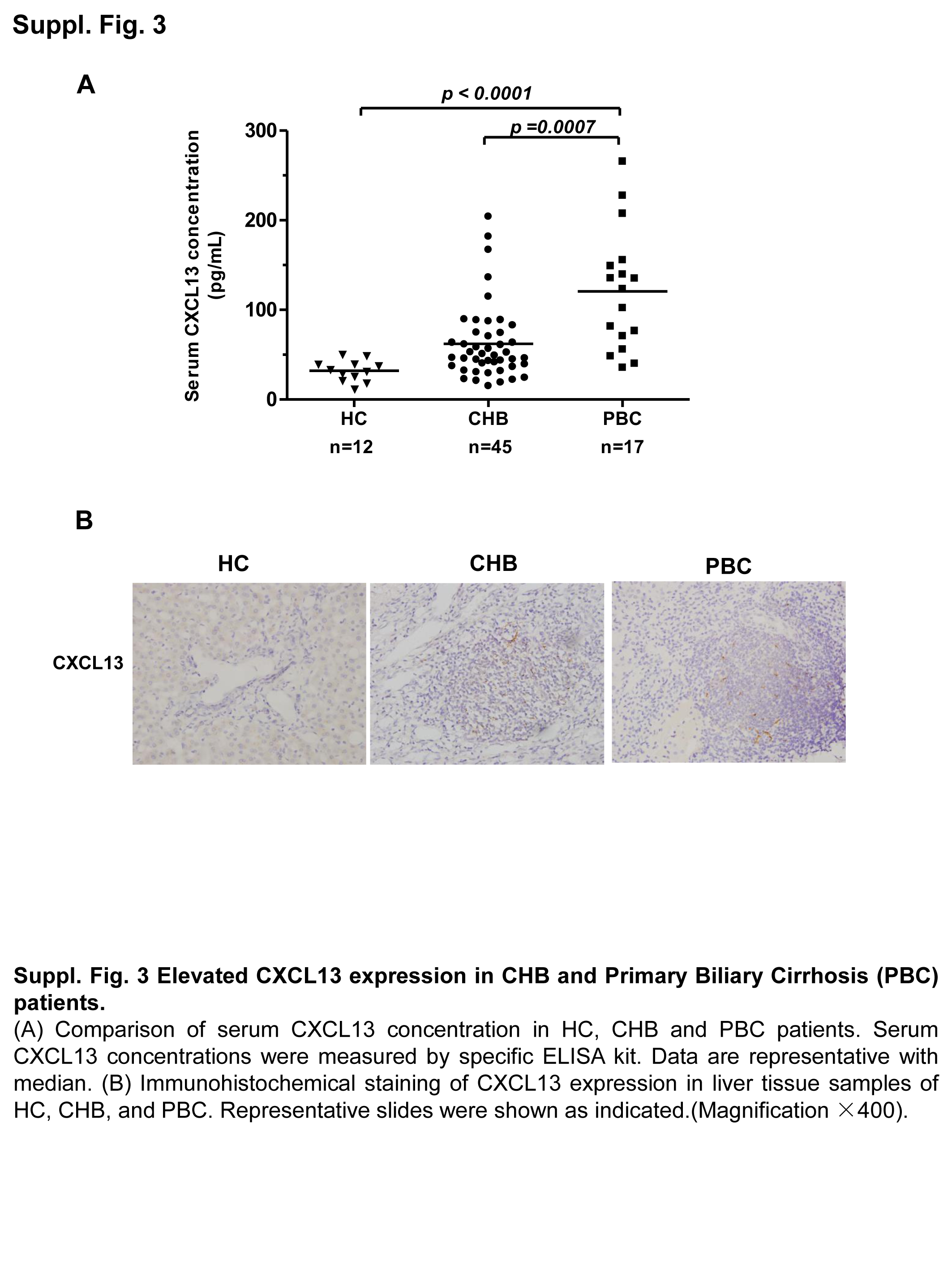

Supplement: Supplementary file 3 [file image_3.tif]
