# Supplementary material for: Elevated Expression of Chemokine CXCL13 in Chronic Hepatitis B Patients Links to Immune Control during Antiviral Therapy
Source: Front Immunol. 2017 Mar 23;8:323. doi: 10.3389/fimmu.2017.00323 (PMC5362616; doi:10.3389/fimmu.2017.00323)
Supplement: Supplementary file 4 [file table_1.docx]

**Supplementary Table 1** **Clinical characteristics of the Liver specimens**

| GROUP | HBV-negative | HBV-positive |
| --- | --- | --- |
| No. of patients | 6 | 6 |
| Gender(M/F) | 2/4 | 3/3 |
| Age(years) | 54.00 ± 5.42 | 60.00 ± 3.69 |
| ALT(U/L) | 322.00 ± 69.87 | 21.50 ± 9.64 |
| AST(U/L) | 305 ± 77.43 | 66.50 ± 16.53 |
| Fibrosis Stage | 2.30 ± 0.40 | 3.10 ± 0.50 |
| HCC (n, %) | 2(33.3%) | 1(16.7%) |
| Liver transplantation  (n, %) | 0(0%) | 5(83.3%) |

Note：Data were shown as median ± SEM
